# Crohn's Disease Associated with Sweet's Syndrome and Sjögren's Syndrome Treated with Infliximab

**DOI:** 10.1080/17402520500134254

**Published:** 2005-06

**Authors:** Erina N. Foster, Khanh K. Nguyen, Rafiq A. Sheikh, Thomas P. Prindiville

**Affiliations:** 1 Division of Gastroenterology and Hepatology University of California at Davis Medical Center Sacramento CA USA; 2 Department of Gastroenterology and Hepatology South Sacramento Kaiser Hospital Sacramento CA USA

## Abstract

The association of Crohn's disease (CD) and Sweet's syndrome is rare and the presence of Sjögren's syndrome in Crohn's disease is even rarer, with only three reports found in the literature. We describe two cases of Crohn's disease associated with Sweet's syndrome, one of which is the first case of CD and Sweet's concomitantly associated with Sjögren's syndrome. Both cases responded rapidly to Infliximab therapy with complete resolution of the skin lesions.


*Clinical & Developmental Immunology*, June 2005; 12(2): 145–149

Taylor & Francis

Taylor & Francis Group

### Crohn's disease associated with Sweet's syndrome and Sjögren's syndrome treated with Infliximab

ERINA N. FOSTER[1], KHANH K. NGUYEN[1], RAFIQ A. SHEIKH[2], & THOMAS P. PRINDIVILLE[1]

[1]*Division of Gastroenterology and Hepatology, University of California at Davis Medical Center, Sacramento, CA, USA, and* [2]*Department of Gastroenterology and Hepatology, South Sacramento Kaiser Hospital, Sacramento, CA, USA*

**Abstract**

The association of Crohn's disease (CD) and Sweet's syndrome is rare and the presence of Sjögren's syndrome in Crohn's disease is even rarer, with only three reports found in the literature. We describe two cases of Crohn's disease associated with Sweet's syndrome, one of which is the first case of CD and Sweet's concomitantly associated with Sjögren's syndrome. Both cases responded rapidly to Infliximab therapy with complete resolution of the skin lesions.

**Keywords:** *Crohn's disease, Infliximab, Sjögren's syndrome, Sweet's syndrome*

### Introduction

Robert Douglas Sweet first described acute febrile neutrophilic dermatosis, or Sweet's syndrome, in 1964. The disorder is characterized by fever, leukocytosis, acute eruption of painful erythematous plaques and nodules on the face, neck, upper chest, back and extremities. Other skin manifestations such as vesicles, pustules, purpura, ulcers and hemorrhagic lesions have been described. The overall female to male ratio is 3.7:1 with the mean age of 52 years. Su and Liu (1986) proposed two major and several minor diagnostic criteria for Sweet's syndrome (Table I). Von den Driesch (1994) added elevated erythrocyte sedimentation rate as a fifth minor criteria. For a definitive diagnosis, both major and two minor criteria must be met. Clinical manifestations are primarily dermatological, although acute neutrophilic alveolitis, pleuropericardial effusion, acute renal failure, aortitis, elevated transaminases and aseptic meningitis have been described. The histologic findings consist of diffuse, predominantly neutrophilic dermal infiltrate with leukocytoclasis, a marked vasodilatation and swelling of the vascular endothelium. The pathogenesis of Sweet's syndrome is poorly understood and several mechanisms have been implicated. There are conflicting reports of local deposits of immunoglobulin and complement components on the vessel wall, although leukocytoclastic vasculitis and intravascular microthrombi are not considered features of Sweet's syndrome (von den Driesch 1994).

Cohen et al. suggested that the production of cytokines, such as G-CSF, interleukin (IL)-1, IL-6, or IL-8, if deposited in the dermis, might be responsible for the immunopathologic and clinical manifestations (Cohen et al. 1992, Reuss-Borst et al. 1993). The fact that Sweet's syndrome can occur after G-CSF treatment shows that IL-1, which is produced by acute myelocytic leukemia (AML) cells and stimulates the G-CSF gene, plays a role in the pathogenesis of Sweet's syndrome (Park et al. 1992, Griffin et al. 1993).

Many diseases with dermatologic manifestations may resemble Sweet's syndrome. Erythema nodosum can at times be almost indistinguishable from Sweet's syndrome. Beside its association with Crohn's disease, Sweet's syndrome has been recognized with many other diseases, including infections, hemoproliferative

Correspondence: T. P. Prindiville, Department of Gastroenterology and Hepatology, University of California, 4150 V. Street Suite 3500 PSSB, Sacramento, CA 95817, USA. Tel: 1 916 734 7183. Fax: 1 916 734 7908.

ISSN 1740-2522 print/ISSN 1740-2530 online © 2005 Taylor & Francis Group Ltd
DOI: 10.1080/17402520500134254

Table I. Revised diagnostic criteria for Sweet's syndrome.

*Major Criteria*
1. Abrupt onset of tender or painful erythematous plaques or nodules occasionally with vesicles, pustules, or bullae.
2. Predominantly neutrophilic infiltration in the dermis without leukocytoclastic vasculitis

*Minor Criteria*
1. Preceded by a nonspecific respiratory or gastrointestinal tract infection or vaccination or associated with:
   Inflammatory diseases such as chronic autoimmune disorders, infections
   Hemoproliferative disorders or solid malignant tumors
   Pregnancy
2. Accompanied by periods of general malaise and fever (>38°C)
3. Laboratory values during onset: ESR > 20 mm, C reactive protein positive, segmented neutrophils >70% in peripheral blood smear, leukocytosis >8000 (3 of 4 of these values are necessary)
4. Excellent response to treatment with systemic corticosteroids or potassium iodide

Both major and two minor criteria are needed for diagnosis.
Taken from reference Su and Liu (1986).

disorders, solid tumors, paraproteinemias, pregnancy, Behcet's syndrome, bowel-bypass syndrome and rarely, vaccination or drug association (von den Driesch 1994).

Only a few cases of Sweet's syndrome associated with Crohn's disease have been reported in the literature. The skin manifestations of Sweets syndrome may parallel the activity of Crohn's disease in some patients (Actis et al. 1995, Fett et al. 1995).

Prednisone or prednisolone at an initial dose of 0.5–1.5 mg/kg of body weight per day, with gradual taper over 2–4 weeks, represents the standard treatment for Sweet's syndrome (von den Driesch 1994). Kemmet et al. (1990) demonstrated that 10% of their patients had chronic relapsing disease for at least 3 years. In recurrent disease, therapy with colchicine, potassium iodide, dapsone, doxycycline, nonsteroidal anti-inflammatory agents and cyclosporine have been described with mixed results (Waltz et al. 1999).

Sjögren's syndrome is an autoimmune disease of unknown etiology affecting predominantly the salivary, lacrimal and exocrine glands. Dry eyes and dry mouth characterize primary Sjögren's syndrome, also called the Sicca syndrome. Secondary Sjögren's syndrome is associated with connective tissue diseases especially rheumatoid arthritis (von den Driesch et al. 1989, Bartunkova et al. 1999). The disease affects approximately 0.2% of adults, mainly older women with a female to male ratio of 10:1 (Bartunkova et al. 1999). Vitali et al. (1993) proposed diagnostic criteria for primary Sjögren's syndrome (Table II). Recent modification of the diagnostic criteria requires the presence of antibody to SS-A or characteristic findings on minor salivary gland biopsy. The pathophysiology of Sjögren's syndrome is characterized by replacement of initial B-lymphocytes and periductal infiltration with T-lymphocytes, resulting in glandular and ductal atrophy. However, the infiltrate does not extend

Table II. Preliminary criteria for the classification of Sjögren's syndrome.

1. Ocular symptoms
*Definition*: A positive response to at least one of the following 3 questions:
   a. Have you had daily persistent, troublesome dry eyes for more than 3 months?
   b. Do you have a recurrent sensation of sand or gravel in the eyes?
   c. Do you use tear substitutes more than 3 times a day?

2. Oral symptoms
*Definition*: A positive response to at least one of the following 3 questions:
   a. Have you had a daily feeling of dry mouth for more than 3 months?
   b. Have you had recurrent or persistently swollen salivary glands as an adult?
   c. Do you frequently drink liquids to aid in the swallowing dry foods?

3. Ocular signs
*Definition*: objective evidence of ocular involvement, determined on the basis of a positive result on at least one of the following 2 tests:
   a. Schirmer-I test (≤ 5 mm in 5 minutes)
   b. Rose bengal score (≥ 4, according to the van Bijsterveld scoring system)

4. Histopathologic features
*Definition*: Focus score ≥1 mm on minor salivary gland biopsy (focus defined as an agglomeration of at least 50 mononuclear cells; focus score defined as the number of foci 4 $mm^2$ of glandular tissue)

5. Salivary gland involvement
*Definition*: objective evidence of salivary gland involvement, determined on the basis of a positive result on at least one of the following 3 tests:
   a. Salivary scintigraphy
   b. Parotid sialography
   c. Un-stimulated salivary flow (≤1.5 ml in 15 minutes)

6. Autoantibodies
*Definition*: presence of at least one of the following serum autoantibodies:
   a. Antibodies to Ro/SS-A or La/SS-B antigens
   b. Antinuclear antibodies
   c. Rheumatoid factor

*Exclusion criteria*: preexisting lymphoma, acquired immunodeficiency syndrome, sarcoidosis, or graft-versus-host disease.

Taken from reference Vitali et al. (1993).

beyond the gland capsule and the normal lobular pattern and salivary ductal epithelium tend to persevere.

Sjögren's syndrome may involve many organs, but oral manifestations are the most common. The patients have symptoms of sensitivity to acidic and spicy foods, difficulty in eating dry foods and difficulty in speaking and controlling dentures (von den Driesch et al. 1989, Soto-Rojas et al. 1998). Oral examination findings include dental caries, angular cheilitis, fissure-lobulated smooth tongue and oral candidiasis (Soto-Rojas et al. 1998). Salivary gland enlargement can occur in two-thirds of patients. Ocular involvement is common and manifests with sensations of grittiness, itching, dryness of the eyes, eye fatigue and photosensitivity. Deficiency of mucin and lipid production leading to decreased tear viscosity and increased tear evaporation are the most important pathophysiological factors for ocular symptoms (Jones et al. 1998, Shimazaki et al. 1998). Other clinical manifestations include, myositis, arthralgias, vaginal dryness, interstitial lung diseases, cystitis and peripheral neuropathy. Sjögren's syndrome has been associated with rheumatoid arthritis, lymphoma, Waldenström's macroglobulinemia, thyroiditis, primary biliary cirrhosis, pernicious anemia and hepatitis C (Ramos-Casals et al. 1998, 1999).

Although the role of viral infections, environmental conditions and predisposing genetic factors all have been suggested, the pathogenesis of Sjögren's syndrome remains unknown. Recruiting T-cells and clonal expansion with release of cytokines like TNF-alpha and IL-1 are believed to interfere with the neural signals, causing inhibition of glandular secretions (Fox et al. 1998, 1999).

### Report of cases

### *Case 1*

A 37-year-old Caucasian female with a four-year history of CD presented with severe sore throat and difficulty in swallowing. Her primary care physician prescribed oral penicillin for the presumptive diagnosis of streptococcal pharyngitis. Three days later, she developed mouth sores forming deep pits and she became febrile to 40°C. She also developed new acneiform pustules on her abdomen, lateral thighs, under her breasts, along her inner labia, face and arms. She was then referred to a dermatologist for evaluation.

Crohn's disease therapy consisted of Infliximab infusions at 5 mg/kg until three months prior to her diagnosis of Sweet's when her dose was increased to 10 mg/kg every six weeks due to the severity of her CD. She had been initially treated with CellCept and 6-Mercaptopurine (6-MP) for her Crohn's disease but developed pancreatitis. Current immunomodulator therapy was methotrexate at 15 mg a week by injection.

Physical examination by the dermatologist revealed 1–1.5 cm round, indurated, erythematous, annular plaques on the right arm and mid abdomen. In addition, scattered acneiform pustules were found in the groin. The uvula was erythematous with a one mm erosion on the right upper inner lip and clustered erosions covered the buccal and gingival mucosa. Laboratory tests were unremarkable except for a white blood cell count of 10,000 with 85% neutrophils. Viral and bacterial throat cultures and HSV immunofluorescence testing from a skin lesion were negative. A punch biopsy from a lesion on the right arm showed histological features of Sweet's syndrome.

Therapy was initiated with indomethacin and was discontinued two days later due to severe nausea. The patient's skin lesions and symptoms did not improve. She was therefore treated with a 10 mg/kg infusion of Infliximab, which led to a complete resolution of her skin lesions within 2–3 days. Subsequent therapy consisted of maintenance Infliximab therapy every 6 weeks and immunomodulation with methotrexate. There has been no recurrence of the skin lesions or the oral ulcers in the last 3 years.

### *Case 2*

A 45-year-old Caucasian female was admitted for severe low back pain after lifting a 75-pound sack. She was treated with intravenous narcotic analgesia with relief of pain. However, the patient developed a fever of 39°C with erythematous tender nodules on her arms and legs requiring transfer to our medical center for further management of presumed erythema nodosum.

She had a 20-year history of CD, multiple small bowel resections and a total colectomy. She was diagnosed with erythema nodosum when similar lesions developed with flares of her CD in past. The patient had been started on 6-MP by her gastroenterologist 2-weeks prior to admission for recurrent small bowel CD. However, prior to transfer, the 6-MP was discontinued and empiric therapy with intravenous broad-spectrum antibiotics was initiated.

Physical examination revealed tachycardia at 120 bpm, normal blood pressure and temperature of 39.5°C. The abdomen was soft, nontender with active bowel sounds, well-healed scars and an intact ostomy bag. There were multiple 1–4 cm poorly marginated, erythematous nodules and few 1–3 mm pustules on her upper and lower extremities. She continued to be febrile and became toxic with worsening of her skin lesions. Laboratory tests revealed a white blood cell count of 11,900/mm$^3$ with 94% neutrophils, hematocrit of 38.5% and platelets 230,000. Urine culture was negative. Blood cultures were not drawn as the patient was transferred on antibiotics. Liver function tests and chemistry panels were normal. A chest X-ray, abdominal and pelvic CT scans were unremarkable. An ileoscopy revealed scattered aphthous ulcers.

She developed photophobia, a gritty feeling in the eyelids, dry and sore mouth with difficulty in swallowing. She also began to develop bilateral firm, tender submandibular swelling extending to involve the parotid glands. Her SS-A, SS-B antibodies, rheumatoid factor and antinuclear antibodies were all negative. Given her classical clinical findings, a clinical diagnosis of Sjögren's syndrome was made by the consulting rheumatologist. She refused a parotid gland biopsy but a punch biopsy of one of the skin lesions revealed dermal neutrophilic infiltration, subepidermal edema and no evidence of vasculitis consistent with the diagnosis of Sweet's syndrome. She was started on prednisone 40 mg daily and experienced significant improvement in her parotid gland enlargement and skin lesions. The patient was then discharged on a tapering course of prednisone. On follow up several weeks later, her parotid enlargement had disappeared but her skin lesions were not improving and she had started to have ostomy site breakdown. Biologic therapy was then started with Infliximab 5 mg/kg by intravenous infusion. The skin lesions completely resolved within one week.

### Discussion

The association of Sweet's syndrome and Sjögren's syndrome with Crohn's disease has not been reported. We found only six case reports of Sjögren's syndrome associated with Sweet's syndrome in the literature, and their association is probably immune-mediated (Bianconcini et al. 1991, Vatan et al. 1997, Osawa et al. 1997). Likewise, Crohn's disease is also an immune-mediated disease that begins with activation of the intestinal immune system. The putative target for this response is directed to the luminal commensal bacteria. The increased mucosal production of TNF-α found in Crohn's disease is a pivotal finding, and neutralization of TNF-α has been shown to be efficacious in the treatment of fistulizing Crohn's disease (Present et al. 1999). Infliximab is a chimeric anti-TNF-α monoclonal antibody, which blocks the binding of TNF-α to its transmembrane receptors (Knight et al. 1993). Additionally, binding is associated with apoptosis of the activated membrane bound T-cells and results in a cellular immune modulation. Literature reviews did not support an association of 6-MP with either Sweet's or Sjögren's syndrome. In a manner similar to Crohn's disease immune activation and resultant cytokine response, Sweet syndrome has reported associations with bacterial infections. We, therefore, believe the development of these syndromes in our patients was associated with the increased activity of the patients Crohn's disease and the resulting increased cytokine response. Andersen and Tiede (1997) described a case of a 43-year-old man, who developed joint pain, fever, rash and serum sickness with acute lobular panniculitis and vasculitis within 2-weeks of 6-MP therapy for CD. Vasculitis is not a feature of Sweet's syndrome and its presence should direct a search for another disease. The rapid response after receiving Infliximab therapy with complete resolution of symptoms and complete resolution of the patient's skin lesions was quite impressive and unexpected. This observation supports the role of TNF-α in the pathogenesis of Sweet's syndrome.

Several studies have supported the role of cytokines in the development of Sweet's syndrome. First, patients with AML, the most common malignant neoplasm associated with Sweet's syndrome, have cells, which produce IL-1 *in vitro* (Griffin et al. 1993). Second, Sweet's syndrome has been described in patients after receiving G-CSF therapy (Park et al. 1992). Finally, high levels of G-CSF and IL-6 have been found in patients during the acute phase of Sweet's syndrome, as opposed to the moderate rise in TNF-α level (Reuss-Borst et al. 1993). Also, TNF-α has been found in the salivary glands of patients with Sjögren's syndrome. These findings, together with the success of Infliximab in the treatment of Crohn's disease imply the potential benefit of anti-TNF antibodies in the treatment of refractory Sweet and Sjögren's syndrome.

### Acknowledgements

Pathology slides prepared by Dr Rafael Rodriguez.

### References

Actis, et al. 1995. Recurrent Sweet's syndrome in reactivated Crohn's disease. J Clin Gastroenterol 21(4):317.

Andersen JM, Tiede JJ. 1997. Serum sickness associated with 6-mercaptopurine in a patient with Crohn's disease. Pharmacotherapy 17(1):173–176.

Bartunkova, et al. 1999. Primary Sjögren's syndrome in children and adolescents: Proposal for diagnostic criteria. Clin Exp Rheumatol 17(3):381–386.

Bianconcini, et al. 1991. Sweet's syndrome (acute febrile neutrophilic dermatosis) associated with Sjögren's syndrome. A clinical case. Minerva Med 82(12):869–876.

Cohen, et al. 1992. Concurrent Sweet's syndrome and erythema nodosum: A report, world literature review and mechanism of pathogenesis. J Rheumatol 19(5):814–820.

Fett, et al. 1995. Sweet's syndrome: Systemic signs and symptoms and associated disorders. Mayo Clin Proc 70(3):234–240.

Fox, et al. 1998. Evolving concepts of diagnosis, pathogenesis, and therapy of Sjögren's syndrome. Curr Opin Rheumatol 10(5):446–456.

Fox, et al. 1999. Current issues in the diagnosis and treatment of Sjögren's syndrome. Curr Opin Rheumatol 11(5):364–371.

Griffin, et al. 1987. Secretion of interleukin-1 by acute myeloblastic leukemia cells *in vitro* induces endothelial cells to secrete colony stimulating factors. Blood 70(4):1218–1221.

Jones, et al. 1998. Alterations of ocular surface gene expression in Sjögren's syndrome. Adv Exp Med Biol 438:533–536.

Kemmet, et al. 1990. Sweet's syndrome: A clinicopathologic review of 29 cases. J Acad Dermatol 23:503–507.

Knight, et al. 1993. Construction and initial characterization of a mouse-human chimeric anti-TNF antibody. Mol Immunol 30(16):1443–1453.

Osawa, et al. 1997. A case of Sjögren's syndrome associated with Sweet's syndrome. Clin Rheumatol 16(1):101–105.

Park, et al. 1992. The Sweet syndrome during therapy with granulocyte colony stimulating factor. Ann Intern Med 116 (12 pt 1):996–998.

Present, et al. 1999. Infliximab for the treatment of fistulas in patients with Crohn's disease. N Engl J Med 340(18):1398–1405.

Ramos-Casals, et al. 1998. Cryoglobulinemia in primary Sjögren's syndrome: Prevalence and clinical characteristics in a series of 115 patients. Semin Arthritis Rheum 28(3):200–205.

Ramos-Casals, et al. 1999. Sjögren's syndrome and hepatitis C virus. Clin Rheumatol 18(2):93–100.

Reuss-Borst, et al. 1993. Sweet's syndrome associated with myelodysplasia: Possible role of cytokines in the pathogenesis of the disease. Br J Haematol 84(2):356–358.

Shimazaki, et al. 1998. Meibomian gland dysfunction in patients with Sjögren's syndrome. Ophthalmology 105(8):1485–1488.
Soto-Rojas, et al. 1998. Oral manifestations in patients with Sjögren's syndrome. J Rheumatol 25(5):906–910.
Su WP, Liu HN. 1986. Diagnostic criteria for Sweet's syndrome. Cutis 37(3):167–174.
Vatan, et al. 1997. Association of primary Gougerot-Sjögren syndrome and Sweet syndrome. Apropos of a case (letter). Rev Med Intern 18(9):734–735.
Vitali, et al. 1993. Preliminary criteria for the classification of Sjögren's syndrome. Results of a prospective concerted action supported by the European community. Arthritis Rheum 36(3):340–347.
Waltz, et al. 1999. Sweet's syndrome and erythema nodosum: The simultaneous occurrence of 2 reactive dermatoses. Arch Dermatol 135(1):62–66.
von den Driesch P. 1994. Sweet's syndrome (acute febrile neutrophilic dermatosis). J Am Acad Dermatol 31(4): 535–556, quiz 557–60.
von den Driesch P, et al. 1989. Sweet's syndrome: Clinical spectrum and associated conditions. Cutis 44(3):193–200.

